# Wanted for the Surgeon General’s Library

**Published:** 1875-11

**Authors:** 


WANTED FOR THE SURGEON GENERAL'S LIBRARY.—In order to complete the file of the *Southern Medical and Surgical Journal*, the following numbers are required: Vol. III, (1838-9); No. 1 of Vol. III, new series (1847); Nos. 1, 2, 3, 4, of Vol. VI; No. 1, Vol. VIII; Nos. 11, 12, Vol. XVII; Vol. XVII; Vol. XVIII.

Liberal prices will be paid for any of the above, and owners are invited to correspond with Dr. John S. Billings, U. S. A., Surgeon General's office, Washington, D. C.

